# Supplementary material for: Are carbon emissions trading and green financial instruments synergistic? -Comprehensive quantitative research based on content analysis
Source: PLoS One. 2024 Mar 7;19(3):e0298601. doi: 10.1371/journal.pone.0298601 (PMC10919601; doi:10.1371/journal.pone.0298601)
Supplement: S1 Data — (ZIP) [file pone.0298601.s001.zip › Data/地级市Z_F工作报告.zip!/╣π╢1⁄2/╒╪╟∞╩╨/└┤╫╘╣1⁄2╓┌║┼í╛┬φ┐╦╩2╛▌═°í┐_2010.docx]

各位代表：

我代表肇庆市人民政府向大会作政府工作报告，请予审议。并请政协委员和其他列席人员提出意见。

危中抢机，经济社会发展成效显著

过去的一年，是我市深入贯彻落实科学发展观，积极应对国际金融危机，全力推进“三促进一保持”，经济社会发展取得显著成效的一年。面对严峻复杂的经济形势，我们在省委、省政府和市委的正确领导下，在市人大及其常委会、市政协的监督支持下，全面贯彻落实中央和省保增长保民生保稳定的各项决策部署，紧紧抓住实施《珠江三角洲地区改革发展规划纲要》（下称《规划纲要》）的历史机遇，解放思想，积极谋划，锐意进取，迎难而上，坚定不移抗危机，全力以赴促发展，脚踏实地惠民生，全市经济社会保持平稳较快发展态势，全面完成或超额完成了市十一届人大四次会议确定的目标任务。

——预期目标全面实现。全市生产总值846.34亿元，比上年增长（下称增长）13.6%，超过预期目标1.6个百分点，比全省平均增幅高4.1个百分点；人均生产总值22008元，增长11.6%；一、二、三产业增加值分别增长5%、19.7%和12.7%。

——工业生产快速发展。规模以上工业增加值277.47亿元，增长22.7%，增幅列全省第三；预计工业利润和税金分别增长37.3%和33.1%。

——特色农业增产增收。农业总产值272.94亿元，增长6%；粮食总产量创历史新高；柑桔、蔬菜、生猪、淡水养殖产量均居全省前列。

——招商引资成效显著。新批外商投资项目82宗，合同投资总额15.2亿美元，实际吸收外资8.88亿美元，增长3.5%。

——重大项目稳步推进。二广高速公路三水至怀集段工程大部分已完成，广佛肇城际轨道肇庆段正式动工；22个列入省重点计划的项目完成年度投资计划135.2%；93个市重点项目完成年度投资计划128.6%；完成全社会固定资产投资462.77亿元，增长28.2%。

——城市建设进程加快。肇庆城东新区二期道路工程顺利完成，星湖大道、信安大道、砚都大道等主干路的改造建设加快推进。

——区域经济协调发展。经济中心区生产总值增长15.3%，山区生产总值增长13.4%。

——财政实力持续增强。地方财政一般预算收入达55.92亿元，增长28.4%，三年翻一番，增幅列全省第四。

——金融服务力度加大。全市金融机构本外币各项贷款余额达568.31亿元，比年初增长51.9%，增幅列全省第三，存贷比为61.7%，创历史新高。

——环保节能取得实效。万元GDP能耗比上年下降4.8%，单位工业增加值能耗下降10.07%，完成年度主要污染物减排任务。

——普高目标提前实现。高中阶段教育毛入学率达86.3%，顺利通过省普及高中阶段教育督导验收，提前两年实现目标。

——人民生活不断改善。城镇居民人均可支配收入15063元，农村居民人均纯收入6291元，分别增长10.4%和7.1%。

——综治维稳切实加强。连续三届获得全国综治优秀市称号，荣获全国综治“长安杯”。

去年我市经济社会发展成效显著，亮点纷呈，这是市委正确领导的结果，是各级、各部门和全市人民团结拼搏、攻坚克难、务实创新的结果。主要工作和成效有：

一、着力推进“三促进一保持”，经济发展态势良好

应对危机措施有力。出台一系列政策措施帮扶企业，积极争取上级批准降低土地使用税征收标准，阶段性下调企业社会保险费部分险种费率和养老保险金企业征缴比例，扩大失业保险金适用范围，减免或缓收部分行政性收费；开展了全市500家企业大调研活动，引导企业“抱团过冬”，主动帮扶企业解决困难和问题，市财政筹集资金4000多万元，专门用于增加中小企业信用担保、开展招商引资、开拓出口市场，全市企业整体生产经营保持稳定；蓝带啤酒、风华高科、星湖科技三大重点企业引入战略投资者取得实效；外贸进出口总额33.7亿美元，降幅低于全省平均水平。

产业结构优化升级。工业总量壮大，结构不断优化，180多个工业重点项目建成投产或达标达产，新增产值80多亿元，规模以上工业总产值首超千亿；电子信息、汽车配件、生物制药、金属材料、新型建材、林产化工等六大重点产业产值占规模以上工业总产值的51.2%；加快传统产业改造提升，关停落后产能企业17家、治理33家，完成工业技术改造投资32亿元，增长38.1%；名牌带动和企业上市战略扎实推进，风华高科和星湖科技的商标被认定为中国驰名商标，实现了我市中国驰名商标“零”的突破，高要天龙油墨上市申请已获国家证监会批准，高新区科茂林产化工等已结束上市辅导期。现代服务业和港口物流业加快发展，《肇庆港总体规划》通过交通部和省有关专家评审，港口货物吞吐量和集装箱吞吐量分别达到1124万吨和34.6万标箱。现代信息服务业逐步形成，有5家企业7个工程项目被评为省信息化、工业化融合示范工程；中巴软件园成功吸引一批研发机构和软件创意企业入驻，超级计算中心、创意产业工程中心等相继建成；文化产业逐步形成特色，端砚特色文化产业园、古宋城保护与开发利用项目加快推进。农业产业化加快，农村土地经营权流转和集体林权制度改革经验在全省推广，珍贵树种种植规模和发展速度居全国前列；县级以上的农业龙头企业108家，专业合作组织433家，带动30多万农户，产业化经营增收4.3亿元；落实种粮综合直补等惠农政策，发放各类农业政策性补贴2.16亿元，全年粮食总产量110.36万吨，柑桔产量80万吨，蔬菜产量230多万吨，生猪年出栏403.9万头，水产养殖产量32.69万吨，农产品价格回升，农民实现增收。

投资规模不断扩大。重点项目和新增中央投资项目列入国家和省计划数、投资规模、落实融资额均创历史新高。有66个项目列入新增中央投资计划，总投资19.9亿元，年度完成投资5.1亿元；22个列入省重点计划项目和93个市重点项目分别完成投资114亿元、173.91亿元。加强政银合作，新增市本级政府性贷款56.94亿元，主要投入基础设施建设和社会事业发展；全市15家信用担保公司为278家中小企业担保贷款5.78亿元，两家小额贷款公司试点成功，城乡各类创业贷款规模不断扩大，促进了地方经济发展。

扩大内需效果明显。认真落实家电、汽车、摩托车、农机下乡以及家电以旧换新、小排量汽车税率下调等政策，城乡消费大幅增长，社会消费品零售总额278.71亿元，增长19.2%；积极组织“肇货北上西进”，在国内重点城市举办各种促销活动，签订贸易合同120多亿元；认真落实房地产业发展政策，完成房地产投资60.66亿元，增长11.7%；商品房销售面积和销售额分别达275.31万平方米和93.49亿元，分别增长83.7%和112.7%；积极推行国民旅游休闲计划，加大旅游宣传促销，旅游景区（点）接待游客1603万人次，旅游收入79.5亿元，分别增长18.1%和20.8%，旅游接待人数和旅游收入增幅均居珠三角九市首位。

二、着力贯彻落实《规划纲要》，招商引资和“双转移”成效显著

实施《规划纲要》初见成效。制定贯彻落实《规划纲要》的实施意见，召开全市动员大会和工作现场会；广佛肇经济圈建设扎实推进，联合召开三市工作座谈会和首次广佛肇市长联席会议，签署三市经济建设合作框架协议和交通运输、产业协作、旅游一体化等一系列合作协议，确定2009-2010年度重点工作计划第一批37个项目；重点推进与广佛地区交通基础设施“无缝对接”，促进广佛地区产业结构调整和扩张延伸项目向我市转移，重点产业基地建设有效推进；整合推出“广佛肇旅游一卡通”，实现与珠三角城市的异地缴存住房公积金贷款合作；高要市与高明区、四会市与三水区、端州区与海珠区建立起结对友好共建关系。

招商引资和“双转移”效果突出。合同利用外资和实际吸收外资再创新高；产业转移园区载体建设扎实推进，肇庆高新区累计融资38.55亿元投入开发建设，基础配套设施更加完善，进园企业累计达500多家，已建成投产150多家；顺德龙江（德庆）、中山大涌（怀集）产业转移工业园建设稳步推进，各县（市、区）工业园区建设水平不断提高；全面加强与珠三角核心区的产业对接和招商引资，全市共引进产业转移项目230个,计划总投资272.43亿元；引进央企取得突破，在北京举办的“肇庆市投资环境推介会”签约项目18宗，合同投资总额231.16亿元；第七届“肇庆金秋”经贸洽谈会共有159宗项目签约、奠基（开工）或剪彩（投产），投资总额537.32亿元；全面推进农村劳动力技能培训和转移就业，共培训农村劳动力38798人，转移农村劳动力52573人，均超额完成年度目标任务。

重大项目建设取得新进展。封开华润水泥一期、高要北新建材等一批工业重点项目建成投产，国电大旺热电联产项目配套工程已开工，四会亚洲金属资源再生工业基地完成一期开发并进入二期建设、志高家电研发基地已进行首期开发建设，广宁燃料乙醇项目已完成50万亩木薯基地规划，封开铜钼矿已列入省有色金属振兴规划和省重点预备项目；鼎湖砚阳湖综合开发项目规划建设正式启动，端州龟顶新城“三旧”改造项目规划建设已经开展；南广、贵广铁路肇庆段全面动工，广肇高速公路二期、珠外环高速公路江门至肇庆段、广梧高速公路封开连接线、国道321线小湘至马房段升级改造、景丰联围除险加固达标等工程建设全面铺开。

生态环保建设扎实推进。积极推行清洁生产，大力发展循环经济，肇庆高新区被列为省循环经济试点园区，新增4家省清洁生产企业；严格执行项目环评和污染排放前置审核制度，加强对重点污染源的管理和实时在线监控；四会独水河综合整治效果明显并成功获得省摘牌；新建成8座污水处理厂，实现“一县一厂”目标；完成第一次全国污染源普查；全市省控主要江河断面水质达到国家地表水Ⅱ类水质标准，饮用水源地水质100％达标，城区空气环境质量达到国家二级标准；注重土地资源保护和节约用地，顺利通过国家和省的卫片执法检查验收；全市森林覆盖率提高到67.4%，新创建绿色学校76家、生态示范村122个、生态示范乡镇2个，端州、怀集成为“广东省林业生态县”；顺利通过国家园林城市复查。

三、着力实施区域协调发展，城乡经济发展不断加快

中心区带动能力增强。城市规划工作加强，完成编制《肇庆市中心区域协调发展规划》，各区、市编制了控制性详细规划；肇庆高新区充分发挥省首批示范性产业转移园的品牌效应，加快项目引进和建设，主要经济指标继续保持高速增长，GDP增长31.2%，地方财政一般预算收入增长52%，实际吸收外资3.1亿美元，继续居全省97个开发区前列；端州区城市经济快速发展，服务业增加值占GDP 63%，电子信息和装备制造业产值占全区工业总产值的76.1%，税收超千万元的企业达19家，人均GDP、人均财政一般预算收入、农民人均纯收入继续保持全市最高水平；鼎湖区重大项目谋划和招商引资有新成效，广新外贸集团、华润集团等大企业成功落户，基础设施建设和城市建设加快推进，商品房销售面积和销售额分别增长504%和488%；四会市经济保持快速发展，地方财政一般预算收入突破8亿元，引进了世界500强企业ABB集团等一批大项目，固定资产投资总额居各县（市、区）首位；高要市实施工业化与城市化并举成效显著，工业税收大幅增长，税收超千万元企业有9家，地方财政一般预算收入10.78亿元，用三年时间实现从3亿元到10亿元的跨越，成为全省首个财政收入超10亿元的山区县（市），加快城市化带动人口增长拉动消费的经验做法得到省委、省政府的充分肯定。

山区经济发展加快。各山区县充分发挥资源优势，大力发展特色产业，推动经济加快发展。广宁县帮助企业“抱团过冬”，稳定纸业生产，林浆纸一体化产业集群被确定为省产业集群升级示范区，地方财政一般预算收入增长36.01%，增幅居各县（市、区）首位；德庆县全力推动三大产业协调发展，地方财政一般预算收入实现三年翻番；封开县大力推动重大项目建设，固定资产投资和实际吸收外资增幅均居全市首位，财政综合增长率在全省67个县（市）中排名第一；怀集县利用特色资源加快发展产业，税收超2000万元的企业达5家。

新农村建设水平提高。全面开通村务信息公开平台，我市被评为全国农村信息化领先地区；推进农村基础设施建设，完成通500人以上大自然村道路硬底化810公里，在全省率先完成建设任务，改造县通镇上等级公路164.15公里；加强农田水利基本建设，争取到省以上水利基础设施建设资金7.73亿元，全面完成机电排灌议案建设任务，城乡水利防灾减灾工程、病险水库除险加固工程加快推进，解决了30万农村人口饮水不安全问题；新建和改造省级农家店185家、省级配送中心2个，进一步完善了农村流通网络体系；推进村庄规划编制试点和农村泥砖住房改造试点；创建省卫生镇3个，新建省卫生村477个、生态文明村506个，累计分别建成1473个和2740个，居全省第一；广宁县被评为全国新农村建设示范县。

四、着力改善民生民利，和谐社会建设扎实推进

民生工程建设加快推进。加大财政对民生重点领域的投入，市、县两级财政对科技、教育、医疗卫生、社会保障和就业、环境保护、农林水事务的支出大幅增长，投入“五大民生工程”资金达6.16亿元，增长33.5%；实施下岗失业人员再就业培训3976人、农民工技能提升培训15275人和创业培训2554人，城镇新增就业人数48648人；常住农业人口新农合参保率100%，补贴标准提高，全年享受新农合补偿234.84万人次，补偿总金额达3.48亿元；乡镇卫生院改造和村卫生站规范化建设加快推进，全市规范化卫生站行政村覆盖率达100%；加强保障性住房建设，新增廉租住房174套、在建337套，新增经济适用住房74套、在建661套；市第一人民医院新院、市中医院新住院楼、市图书馆等重点公共服务项目开工建设。

社会保障能力不断增强。着力创建省统筹城乡就业示范市和国家级创业型城市，城镇登记失业率为2.39%，控制在省规定指标以内；社会保险五大险种参保人数和城镇居民医疗保险参保登记人数完成省下达任务；按时足额发放养老、失业、医疗、工伤和生育保险金，企业退休人员人均养老金增长21.6%；建立了城镇基本医疗保险门诊统筹；积极探索新型农村养老保险制度，端州区被征地农民养老保障试点覆盖面参保率达92.28%，其中强制性参保率100%，均居全省试点县（区）前列；德庆县开展了全省新型农村社会养老保险试点工作；完善城乡居民最低生活保障制度，对低保对象实行分类施保，向低收入家庭增发临时性生活补贴，对低收入困难群体实施了公用事业价格优惠政策。

扶贫、援川工作扎实开展。建立落实“规划到户、责任到人”的扶贫工作机制，积极配合省直部门和佛山市的对口帮扶，农村扶贫开发加快推进；援川灾后重建资金全部到位，累计投入1.37亿元，克枯乡小学、卫生院、供水等项目建成交付使用，各项援建任务基本完成。

五、着力发展社会各项事业，公共服务水平不断提高

科教兴市战略加快实施。肇庆市和高要、四会、德庆被评为全国科技进步考核先进市、县；与清华大学签订全面合作协议，与近百家高校及科研院所开展技术合作；出台实施引进和培养高层次人才政策，引进硕士以上专业人才98人；新认定高新技术企业21家、省高新技术产品52个，实施省级以上科技项目83项，新增省级企业技术中心3家，新组建省级工程技术研究开发中心2家、市级6家，省级技术创新专业镇1个；全市高新技术产业产值151亿元，专利申请量和授权量分别增长31.5%和22.3%。普及高中阶段教育提前两年达标，新建扩建高中阶段学校30所，新增高中阶段学位3万个；创建国家、省级重点职校各1所，职业院校在校生人数达14.23万人，规模居全省前列；着力推进教育均衡发展，完全小学以上义务教育规范化学校达60%以上，农村中小学装备和信息化工程加快推进；乡镇中心幼儿园开办率100%，居全省首位；继续推进“强师振教”工程，611名中小学代课教师转为公办教师，全市中小学教师实现工资福利待遇“两相当”。

文化、卫生、体育工作有效推进。加强文化基础设施建设，完成市粤剧演艺中心、市群众艺术馆改造建设；组织开展第四届读书节，推出了一批文化精品，粤剧《钟馗》等5个作品获省“五个一工程奖”，广播剧《梦想在望》获得全国大奖；开展第三次全国文物普查，崇禧塔景区规划建设和高要学宫维修工程已经启动，5个项目入选第三批省级非物质文化遗产名录，10人获中国文房四宝工艺大师称号，17人获省工艺美术大师称号，端砚被列为广州亚运会特许商品。顺利通过全国创建文明城市先进城市复查。医疗卫生服务体系、疾病防控和卫生应急体系建设加强，有效防控甲型流感等重大传染病，妇幼保健和中医药事业发展加快，无偿献血率达100%；加强卫生监督，完成餐饮业、住宿业和游泳场等公共场所量化分级评审。群众体育活动蓬勃开展，竞技体育再创佳绩，我市健儿在十一届全运会夺得9枚金牌，成绩居全省前列。

人口计生、民政等社会事业持续发展。落实人口计生层级动态管理责任制，全面完成了省下达的人口计生控制计划，端州区被评为全国计划生育优质服务先进单位。实施“敬老福星工程”，资助中心镇敬老院上等级，五保供养水平得到提高，慈善事业得到发展。国防教育和后备力量建设不断加强，基层民兵营（连）“四个基本”达标建设进展顺利，“双拥”工作得到加强。授予了第四批“肇庆市荣誉市民”。民族、宗教、外事、侨务、港澳、妇女儿童、老龄、残疾人、统计、档案、人防、地方志、气象、地震等工作取得新成绩。

六、着力加强服务型政府建设，行政效能进一步提升

民主法制建设得到加强。坚持向人民代表大会及其常委会报告工作、向人民政协通报情况，提交市人大常委会各类专项工作报告22件，实行市领导领办人大、政协建议提案制度，组织办理人民代表建议111件、政协提案164件；密切与各民主党派、工商联、工会、共青团、妇联等的联系；加强行政复议和行政应诉工作；重大事项社会公示和听证制度得到落实；市政府获得省政府授予政务公开工作优秀单位称号；“五五”普法工作深入开展，法治县（市、区）和“民主法治示范村（社区）”创建活动取得实效。

综治维稳工作更加巩固。重视群众来信来访，积极化解社会矛盾；积极做好防范和责任落实，安全生产形势保持稳定；加强社会治安综合治理，整合基层综治、司法、信访资源，加强镇街综治信访维稳中心建设，深入开展严打整治，平安肇庆建设成效显著，被评为全国社会治安综合治理优秀地市、全省维护稳定及社会治安综合治理先进市；封开县“强综治、创平安、促发展”先行试点经验在全省推广。

市场监管力度加大。积极开展产品质量和食品药品安全专项整治行动，加大打击制假售假、走私贩私、非法传销力度；加强招投标监督管理，增强价格调控监管；开展了商务综合行政执法全国试点工作。国税、地税、海关、检验检疫、边检、工商管理、质量技术监督等中央、省驻肇单位为支持地方经济发展作出了积极贡献。

机关作风不断改善。深入开展学习实践科学发展观和作风纪律教育活动；市政府机构改革基本完成，37个部门合并调整为32个；全面落实经济工作责任制考核办法、政府重大投资项目报告制度、政府性固定资产投资管理制度和政府投资项目代建制，完成了第三轮行政审批项目清理工作，行政效能不断提高；政府电子政务系统和政府网站建设不断加强，市政府门户网站“中国肇庆”被评为中国用户满意十佳城市政府门户网站；市行政服务中心搬迁扩展，服务事项增加到300多项；落实党风廉政建设责任制，严格审计监督，严肃查处了一批违法违纪案件；“行风热线”越办越好，市直机关作风评议深入开展。

各位代表，去年市政府工作在困难巨大的环境下顺利完成，是在省委、省政府和市委的正确领导，市人大、市政协的监督支持下取得的，凝聚着全市上下、社会各界的智慧和汗水。在此，我代表市人民政府，向全市广大工人、农民、干部职工、知识分子、外来务工人员、驻肇单位、驻肇部队、武警官兵以及各界人士致以崇高的敬意！向关心和支持肇庆建设发展的外来投资者、港澳台同胞、海外侨胞和国际友人表示衷心的感谢！

在总结成绩的同时，我们也清醒地认识到，我市经济社会发展还存在不少困难和问题：一是经济总量不大、质量有待提高；二是产业竞争力不强，产业结构还不合理；三是重大项目引进仍未突破，重大工业项目缺乏；四是外贸出口仍然困难，出口拉动乏力；五是城市规划建设滞后，城市管理体制仍未理顺；六是城乡居民收入水平有待提高，部分群众生产生活仍面临不少困难；七是机关作风有待改进，政府自身建设须不断加强；等等。我们必须正视存在的困难和问题，采取更加有力的措施切实加以解决。

审时度势，切实把握新的发展机遇

2010年是实施“十一五”规划的最后一年，是我市尽快实现GDP超千亿元、尽快成为名符其实的珠三角城市的关键之年。做好今年的政府工作意义十分重大。从国际国内经济形势分析，今年经济社会发展仍然是机遇与挑战并存，机遇大于挑战。从国际看，全球经济逐步回暖，一些发达国家经济开始复苏。从国内看，国家宏观经济刺激政策效果明显，并继续实行积极的财政政策和适度宽松的货币政策，积极因素在不断增加。从省内看，省委、省政府采取加快转变经济发展方式、深入实施《规划纲要》、“三促进一保持”和“双转移”等一系列战略措施，为我市加快发展提供了强有力的政策支持。从我市看，我们正处在一个重大的战略机遇期，实现科学发展、加快发展具备了许多有利条件：一是省委、省政府为我市发展科学定位。省委、省政府对肇庆寄予厚望，要求我市近期要尽快实现GDP超千亿元、尽快成为名符其实的珠三角城市，远期要成为未来广东发展的新增长极、成为能够代表广东科学发展成果的城市，为我市科学发展指明了方向。二是《规划纲要》深入实施带来先机。肇庆在珠三角一体化发展背景下积极推动“五大对接”，明确六大战略定位，提出了十一项主要任务和肇庆高新区等十三个重点发展载体，使我市未来发展思路更加清晰，后发优势更加凸显。三是广佛肇经济圈建设全面提速。三市已经确定合作发展目标任务，首批重点合作项目已进入实施阶段，必将有力推进肇庆与广佛一体化发展。四是工业发展载体日臻完善。工业园区和产业集聚基地基础设施建设加快推进，为招商引资和承接发达地区产业转移以及央企、省企落户提供了良好载体。五是重大项目带动能力增强。一批重大交通基础设施项目加快建设，一批工业项目建成投产，将成为我市经济加快发展的强大推动力。但是，我们也要清醒地看到，虽然经济形势总体向好，但经济回升的基础尚不稳固，全球经济复苏还有许多不确定因素，国家宏观经济政策所带来的积极效果也需要一个发展过程，经济运行仍存在不少困难和问题。因此，我们既要看到有利因素，坚定发展信心，又要充分认识面临的困难和问题，增强忧患意识，把思想统一到中央、省对当前形势的判断和决策部署上来，统一到市委十届八次全会对今年工作的部署上来，进一步解放思想，把握有利时机，积极谋划科学发展，统筹做好今年政府工作。

今年政府工作的总体要求是：以邓小平理论和“三个代表”重要思想为指导，深入贯彻落实科学发展观和党的十七大、十七届四中全会、中央经济工作会议以及省委十届六次全会精神，按照市委十届八次全会的部署，紧紧围绕尽快实现GDP超千亿元、尽快成为名符其实的珠江三角洲城市的奋斗目标，以加快转变经济发展方式为核心，全面实施《规划纲要》，贯彻落实“三促进一保持”和“双转移”战略。扎实推进经济发展，不断扩大经济规模；扎实推进结构调整，不断提高发展水平；扎实推进县域经济发展，不断缩小区域发展差距；扎实推进城市化进程，不断加快宜居城乡建设；扎实推进民生工程，不断加快社会各项事业发展；扎实推进社会治安综合治理，不断维护社会和谐稳定。全面完成“十一五”规划各项目标任务，为建设繁荣活力、文明法治、和谐安康、生态环保肇庆打下更加坚实的基础。

经济社会发展的主要预期目标是：生产总值增长14%，人均生产总值增长12.6%，一、二、三产业增加值分别增长5%、18%、14.5%；规模以上工业增加值增长22.7%；万元GDP能耗下降、二氧化硫和化学需氧量排放总量减排实现“十一五”目标；全社会固定资产投资增长20%；地方财政一般预算收入增长15%；社会消费品零售总额增长19.5%；合同外资、实际吸收外资分别增长3%以上；外贸进出口增长5%；居民消费价格涨幅控制在3%左右；服务业增加值占GDP比重达42.5%；全社会R&D投入占GDP比重0.97%；城镇登记失业率控制在3%以内；人口自然增长率控制在7.2‰以内；城镇居民人均可支配收入增长9%，农村居民人均纯收入增长7%。

为了实现上述目标，我们将采取扎实措施，全力以赴加快发展，做到抓住一个核心，实现五个新突破。一个核心：就是加快转变经济发展方式，这是今年经济工作的首要任务。要认真贯彻落实胡锦涛总书记在省部级主要领导干部深入贯彻落实科学发展观加快经济发展方式转变专题研讨班上的重要讲话精神，把加快经济发展方式转变作为深入贯彻落实科学发展观的重要目标和战略举措，毫不动摇地加快经济发展方式转变，不断提高经济发展质量和效益，加快推进经济社会协调发展，推动我市走上生产发展、生活富裕、生态良好的文明发展道路。五个新突破：一要在壮大总量、扩大规模上有新突破。科学发展观的第一要义是发展。加快发展、做大经济总量是肇庆目前面临的主要任务。要坚定不移实施“五大发展战略”，加速发展“四大经济”，积极扩内需，促消费，保增长，尽快做大总量、壮大实力。二要在调整结构、促进转型上有新突破。加快发展要摒弃传统的粗放经营式发展，切实加快经济发展方式转变，在调整产业结构、产品结构、需求结构、区域经济结构、外贸出口结构、土地开发结构和收入分配结构等方面取得成效，努力开辟一条经济社会又好又快发展的新路子。三要在扩大招商、引进项目上有新突破。坚定不移推进“双转移”战略，积极谋划建设投资规模大、带动能力强的重大项目，建设布局合理、功能完善、各具特色的产业园区，千方百计引进创税型、科技型、规模型、环保型项目。四要在推进改革、创新体制上有新突破。全面推进大部制改革、富县强镇事权改革、国企改革、林权制度改革、文化及旅游体制改革，探索和建立公共服务均等化的体制机制。五要在突出重点、狠抓落实上有新突破。进一步提升政府抓落实的执行力，完善政府工作问责制，强化对重大决策部署、重点工作、重大项目的跟踪督查，真抓实干，务求实效。

务实进取，努力开创政府工作新局面

今年是肇庆实现科学发展、加快发展的关键一年，为确保完成或超额完成今年发展目标任务，必须做好以下十方面工作：

一、全力促进经济又好又快发展

切实加快工业经济发展。编制实施重点支柱产业发展规划，积极培育肇庆工业50强，力争多家企业进入广东现代产业500强。改造提升传统优势产业，做大做强做优产业集群。推进高要金利小五金、广宁林浆纸一体化省级产业集群升级示范区建设，创建电子元器件、精密压铸、金属加工等省级产业集群升级示范区。加快发展先进制造业和高新技术产业。大力扶持发展一批先进制造企业，推进省市共建汽车零部件产业基地建设；以中巴软件园为龙头带动发展软件产业、文化创意产业，做大做强肇庆国家级电子信息产业基地。推进以星湖科技为龙头的生物医药与健康产业基地建设，启动粤台光电产业园规划建设，支持发展新能源电动车产业、环保产业和新材料产业。积极谋划和加快建设重大工业项目。全力促进亚洲铝业二期、华润水泥一期等重大项目达标达产，推进国电大旺项目一期、四会亚洲金属资源再生工业基地二期、志高家电研发基地等重大工业项目建设。加大对中小企业扶持力度，缓解中小企业融资难、招工难等问题。实施品牌创建和上市发展战略，再创一批国家级、省级名牌产品和驰（著）名商标，在高要天龙油墨成功上市的基础上，再争取1-2家企业上市融资。

着力培育发展现代服务业。大力发展城市经济，支持发展金融服务、文化创意、电子商务、连锁经营、服务外包等新兴服务业，培育发展“总部经济”。推进“物联网”和无线城市建设。做强做优旅游产业，加快整合旅游资源，完成星湖风景名胜区总体规划修编，加快七星岩景区升级改造和东门广场建设，开展鼎湖山新入口规划、立项工作，大力开发中高端旅游产品。积极建设广佛肇优质生活圈，提升商住小区开发建设水平，支持居民自住和改善型住房消费，促进房地产业持续健康发展。实施《肇庆港总体规划》，加快肇庆新港扩建及临港物流园区、海关特殊监管区建设。

大力推动自主创新。加大财政性资金对自主创新支持，积极引进高新技术产业风险投资基金，带动企业增加自主创新研发投入，不断提高科技成果集聚转化能力。深化校市合作和产学研合作，支持企业与高校、科研院所重点实验室共建分支机构，加强产学研创新联盟、示范基地建设，争取一批重点创新项目进入全省自主创新100强，培育一批高新技术企业，支持企业创办博士后工作站、高技能人才培养工作站和研发中心。积极引进各类创新人才，充分发挥科技特派员作用。大力实施知识产权战略，加快专利技术产业化进程，加大知识产权保护力度。

扎实做好财税金融工作。大力培植财源税源，强化财税管理，支持财税部门严格依法征收，努力提高财政收入质量。完善公共财政体系，重点保运作、保民生、保稳定、促发展。继续调整优化支出结构，从严控制一般性支出增长。深化财政管理体制改革，落实政府性贷款“借用还”机制，切实防范政府财政金融风险。优化金融生态环境，扩大组建政府性融资平台，拓宽政银企合作领域。积极推进端州区、高要市农村信用合作社改组为农村商业银行。引导金融机构加大对地方经济社会发展的支持，加快发展中小企业信用担保机构，扩大小额贷款公司试点，努力破解中小企业和“三农”领域融资难问题。

大力发展农业农村经济。把扩大农村需求作为拉动内需的关键举措，把发展现代农业作为转变经济发展方式的重大任务。落实强农惠农政策，继续加大“三农”投入，统筹城乡发展，千方百计增加农民收入。加强农业农村基础设施建设，基本完成城乡水利防灾减灾工程和病险水库除险加固工程。积极建设高要国家级现代农业产业园和怀集平原区现代农业示范园，做强做优粮食、柑桔、蔬菜、禽畜、优质水产品五大主导产业。打造西江北岸柑桔优质水果产业带、中心区花卉园艺产业带、珠三角城市蔬菜生产基地、优质水稻产业工程、重点生猪标准化规模养殖示范工程。提高农业产业化水平，完善农业技术培训推广、农业信息化、农产品检测监督、动植物防疫和农业机械化五大服务体系，推进农业标准化生产，增强农业龙头企业带动能力，促进农户增收。大力发展现代林业，积极创建国家级现代林业试验示范区，扩大珍贵树种种植规模。

二、积极推进广佛肇一体化发展

全面落实合作项目。积极配合省编制珠三角五个一体化专项规划。全面落实首次广佛肇市长联席会议议定事项，共同编制广佛肇经济圈发展规划，加快实施广佛肇首批重点合作计划项目，研究出台第二批合作项目。推进广佛肇城际轨道、高速铁路、高速公路建设，重点加强与广州铁路新客站、白云国际机场、南沙港等交通基础设施对接，实现广佛肇车辆通行费年票互通互认。

拓展合作空间和领域。加强与广佛地区产业对接，探索共建产业转移工业园，主动承接产业转移，积极吸纳人流、物流、信息流、资金流等经济发展要素，使肇庆成为广佛肇经济圈的投资热土。拓展合作领域层次，推动政府、企业、社会多层面合作，努力在公共领域协调共治方面取得实质性进展。

加强工作考核督查。建立健全实施《规划纲要》工作检查考核机制，完善考核奖惩办法，重点考核我市《实施意见》确立的十一项主要任务、十三个重点发展载体和二十三项发展指标的落实完成情况，全力推进各项工作。

三、加快园区建设不断扩大招商引资

全面加强产业园区建设。按照建设成为国家级高新区和尽快实现产值5000亿元的目标，加快肇庆高新区建设和发展，推进申报国家级高新区工作，积极扩大融资规模，加快园区基础设施和服务配套设施建设，增强产业集聚功能，把高新区打造成为央企、省企扩张发展的重要基地。德庆、怀集两个产业转移工业园和其他县（市、区）的工业园区，要积极探索市场化手段加快园区开发建设，提高产业承接能力，积极承接发达地区产业转移。

不断加大招商引资力度。继续创新招商机制和方式，瞄准大企业、盯住500强，精心策划和组织好各类经贸洽谈活动，加强对港澳台、美欧、日韩、东盟等国家和地区的招商。强化招商引资目标责任制，落实重大项目促建促产制度，力争引进重大项目和实际吸收外资取得更大突破。

认真抓好“双转移”工作落实。继续实施省“双转移”战略，加强与珠三角核心区的联系沟通，扩大重点产业的引进合作，积极承接优势产业转移。继续抓好职业技能培训，支持配合省开展技工院校“百校扶百县”等活动，大力发展定向培训、订单培训，促进农村劳动力转移就业，加快发展劳务经济。

四、着力谋划推进重大项目建设

积极谋划重大项目。抓好“项目库”建设，做好“十二五”规划重点项目筛选储备。争取国电大旺热电联产等项目尽快获得国家核准，力争肇庆核电及核电装备制造业进入国家“十二五”规划，推动封开抽水蓄能电站进入省和国家的建设规划，争取端砚文化特色产业集聚园、肇庆新港综合物流产业园等项目纳入省落实《规划纲要》的专项规划。积极谋划建设一批规模超百亿、带动能力强的工业项目。

加快建设重点项目。落实重大项目推进联席会议制度，全力以赴抓好在建重点项目的征地、拆迁、动工和促建工作，全力协调解决建设中的困难和问题，切实加快重大项目建设。确保二广高速公路三水至怀集段、广肇高速公路二期、国道321线小湘至马房段升级改造工程、广梧高速公路封开连接线建成通车。全面铺开肇庆至花都高速公路、珠外环高速公路四会黄岗至花都花山段、二广高速公路连州至怀集段和支线怀集至岗坪段建设。加快实施与佛山对接的国道、省道按同标准建设工程。积极配合建设广佛肇城际轨道肇庆段、贵广和南广铁路肇庆段、西江航道肇庆大桥至封开界首段。尽快启动一批新的交通重点项目立项和建设。加快实施省天然气管道工程肇庆段项目。完成景丰联围除险加固达标主体工程。

五、继续扩大内需促进消费

有效扩大消费需求。多渠道增加居民收入，建立完善工资正常增长机制，加大对低收入困难群众帮扶力度，增强居民消费能力。发展和完善消费信贷，进一步改善消费环境。全面落实家电、汽车、摩托车下乡及以旧换新政策，继续组织实施“万村千乡”市场工程，扩大农村消费需求。扩大家电、汽车、住房、旅游、信息服务等重点消费，发展教育培训、家政服务、文化娱乐、体育健身、休闲旅游等消费。继续推动“肇货北上西进”，鼓励支持出口企业拓展内销市场。

促进投资持续增长。全年计划安排重点建设项目115项，年度计划投资220.8亿元。争取更多项目进入新增中央投资计划和省重点项目计划，争取国家、省扩大内需投资项目和财政性扶持资金。高度重视和扶持民营经济发展，落实鼓励民间投资政策措施，争取金融机构增加各类创业贷款，优化投资环境，激活民间投资。优化投资结构，引导各类资金投向基础设施、产业发展、技术改造、生态建设、改善民生等领域。

力促外贸出口稳步发展。加快推进对外经济发展方式转变，加快调整出口贸易结构。全面落实外贸出口扶持政策，支持企业抢抓订单，努力保持大宗优势产品国际市场占有率。实施出口市场多元化，巩固扩大欧美日传统市场，积极拓展东盟、非洲、中东、南美、俄罗斯等新兴市场。引导支持出口企业培育自主出口品牌，增强出口竞争力。

六、切实加快宜居城乡建设

加强城市规划建设。开展“城市建设年”活动，高起点、高标准推进城市规划编制和修编工作，完善和完成一批控制性详细规划，启动编制一批专项规划。严格规划控制，提高规划执行力。加快“东扩南连”步伐，形成“东西呼应、南北互动”大城建格局。配合做好珠三角绿道网工程规划实施，加快我市绿道网建设。加大土地储备力度，积极掌控土地资源，为加快发展提供强有力的保障。推进城市基础设施建设，加快星湖大道、信安大道、砚都大道改造建设和城东新区三期道路工程，启动星湖牌坊广场改造、环星湖栈道一期等工程建设。促进市政设施向鼎湖辐射延伸，加快推进市区一体化发展。大力推进旧城镇、旧厂房、旧村庄“三旧”改造工作，用足、用好、用活“三旧”改造政策，把“三旧”改造与加快城镇化建设相结合，与建设宜居城乡相结合，与推动文化休闲旅游产业发展相结合，与节约集约用地相结合，与扩大内需相结合，加快推进龟顶新城项目等一批“三旧”改造试点，实现一年试点、两年重点突破的目标。

提升城市管理水平。深化城市管理体制改革，理顺端州城区环境卫生管理体制。加强对城市出入口的整治，推进“三清理”（清理查处城区违法建设、恶意抢种抢搭建和非法采石取土）专项行动。完成城区端州路、建设路、肇庆大道和信安大道四条主干道的绿化改造并强化交通严管。实施星湖环境综合整治。加快城市公共交通站场建设，推进中心区城乡公交一体化。逐步缓解端州城区交通拥堵问题。实施农民工积分制入户政策，扩大城区人口规模。

抓好环保和节约用地。落实节能减排问责制和“一票否决制”。开展循环经济园区和低碳经济园区试点。制定实施不同发展功能区产业准入机制，实行经济发展与环境保护相结合。加快端州城区污水管网、县级和中心镇污水处理设施建设，加强大气和河涌污染防治，加快淘汰落后产能，全面完成“十一五”污染减排任务。实施自然村“万村绿”工程，再创一批绿色学校、生态示范村和生态示范乡镇，加快推进沿江防护林建设保护和生态公益林建设工程。完成市、县、镇三级土地利用总体规划修编，积极开发补充耕地，严格落实耕地保护责任，加强土地计划管理，提高节约集约用地水平。加大闲置土地处置力度，严格查处违法违规用地行为。推进矿业权实地核查工作，切实加强矿山动态监管。

七、进一步加快县域经济发展

积极实施富县强镇工程。推进富县强镇事权改革，大力简政放权，转变县镇政府职能，全面落实富县强镇各项工作任务。优化产业发展规划布局，加强沿江沿路产业园区规划，加快产业发展载体建设。加大园区产业招商力度，引进发展新兴产业和改造提升传统产业并举，发展壮大新型建材、林产化工、矿产开发加工等资源深加工产业，做大做强县域特色产业。

努力提高城镇化水平。推进县城和中心镇规划建设，完善道路管网、公共交通和公共服务设施，提高城镇综合承载能力。培育发展一批省级特色主导产业示范专业镇和县镇区域性商贸中心。加强县域旅游资源深度开发和整合，发展以休闲健康度假为特色的旅游产业。大力发展生态型商住小区，提高县域房地产开发水平。逐步放宽城镇落户条件限制，鼓励支持符合条件的农业人口在城镇就业和落户。

深入推进新农村建设。积极创建卫生镇，抓好村庄规划编制，继续创建省卫生村和生态文明村。扎实推进“村村通自来水工程”，切实提高农村自来水普及率和无害化厕所覆盖率，完成中央下达的农村饮水安全工程年度建设任务。继续实施农村危桥改造和乡村道路建设，推进通300人以上自然村道路硬底化工程。

八、不断深化体制机制改革

深化行政体制改革。按照建立大部制的要求，全面推进县级政府机构改革。争取行政区划调整方案尽快获批，抓好端州区“镇改街”和“村改居”工作。深化行政审批制度改革，完善综合行政执法改革。大力推动事业单位人事制度改革，积极实施事业单位绩效工资制度。

深化经济体制改革。配合做好省直管县财政体制改革，积极参与建立“省保县、市保镇、县保村”隔层财政保障机制。深化国有企业体制改革，继续整合市属国有经济，全面完成中小企业改革扫尾工作。加强国有资产监督管理，确保国有资产保值增值，推动市属国有经济新发展。深化投融资体制改革，全面推进核准制和备案制，进一步规范政府投资项目代建制和建设工程项目招投标工作。加大农村综合改革力度，积极探索建立土地经营权流转和宅基地交易制度，全面推进集体林权制度改革。深化旅游体制改革，激活旅游投资市场，增强市级旅游企业开发融资能力，加快景区景点开发建设。

深化文化体制改革。推进经营性文化事业单位转企改制工作，推动与省有线广电网络产权整合重组和新华书店重组。大力推进文化产业投资多元化，加快发展文化产业。抓好乡镇综合文化站建设，鼓励社会力量参与公共文化建设。

九、切实改善民生加快社会事业发展

深入推进民生工程建设。认真组织实施基本公共服务均等化规划。以创建全省统筹城乡就业示范市和国家级创业型城市为抓手，实施农村“一户一技能”和职业技能培训工程，争取成功创业率达80%以上。推进新一轮中小学校布局调整，加快实施中小学校舍安全工程和农村中小学新装备工程建设。建立覆盖城乡居民的基本医疗卫生制度，推进乡镇卫生院危房改造和设备配套工作。抓好保障性住房建设，全市新建廉租住房278套，经济适用住房346套。加快推进农村泥砖住房改造试点。按计划进度完成“规划到户、责任到人”扶贫开发工作任务。推进重大地质灾害危险点勘查治理和搬迁避让工程建设。继续支援汶川克枯乡灾后重建，确保今年9月前全面完成援建任务。

努力提高城乡社会保障水平。以非公有制企业为重点扩大社会保险覆盖面，逐步实施社会保障卡制度。逐步实现养老保险市级统筹，提高企业退休人员养老保险待遇。推进被征地农民就业，落实被征地农民社会保障工作。抓好德庆县新型农村社会养老保险试点。扩大城乡一体化的医疗保险覆盖范围，巩固新农合参保率，推进基本医疗保险普通门诊统筹业务。建立健全救灾救助体系，创建一批国家综合减灾示范社区。完善低保、五保标准动态调整机制，提高五保供养标准。发展普惠型社会福利，不断完善城乡一体化养老服务体系。

繁荣发展社会各项事业。优先发展教育，加快教育强市建设。建立县域内义务教育资源合理配置机制，推进优质高中阶段学校建设，创建一批教育强镇，巩固提高普及九年义务教育成果和普及高中阶段教育水平，推进“强师振教”工程，提高高等教育发展水平，抓好南方重要职教基地建设，重视发展学前教育和特殊教育。积极创建全国文明城市。加快建设文化强市，推进公共文化服务设施建设，抓好市图书馆、文化艺术中心等项目建设，积极实施文化惠民工程，完成一批重点文化景区景点的规划，推进端砚文化景区景点的保护和开发，着力打造端砚文化、宋文化等特色文化品牌，培育文化创意产业，扶持一批重点文化项目和文化骨干企业，继续抓好第三次全国文物普查。巩固国家卫生城市创建成果。推进医疗卫生体制改革，认真实施国家公共卫生服务项目，稳妥开展公立医院改革，发展民营医疗机构，实施基本药物制度，加快医药分开。加快市第一人民医院新院建设，扶持和促进中医药事业，抓好中医名院创建。加强重大疫情监测和预警工作，严格落实传染性疾病防控措施。深入开展群众性体育活动，贯彻落实《全民健身条例》，积极备战广州亚运会和第十三届省运会。完善人口计生工作机制，深入开展“两无”活动，稳定低生育水平，提高出生人口素质，推动人口计生三类镇转化为二类镇，确保全市人口计生工作达到全省中上水平。组织开展第六次全国人口普查。开展创建全国“双拥”模范城市活动。宣传贯彻新颁布的《国防动员法》，完善国防动员体系，做好优抚安置和“双拥”工作，开展“十百千万关爱功臣”活动，支持退伍军人自主创业，推进基层民兵营（连）达标工程建设。争取与更多国外城市结为新的友好城市。鼓励多层次、多渠道、多形式的慈善活动。落实民族和宗教政策。强化殡葬工作责任制。加强外事、侨务、港澳、妇女儿童、老龄、残疾人、统计、档案、人防、地方志、气象、地震等工作。

全力维护社会和谐稳定。加强和改善社会管理，完善社会管理体制机制，推进以“居住证”为核心的流动人口服务管理“一证通”制度。强化落实信访维稳责任，发挥人民调解与司法调解、行政调解衔接联动效应，着力化解矛盾纠纷。完善公益法律援助体系。开展劳动关系和谐企业、劳动关系和谐工业园区创建活动。创新县、镇、村三级综治管理，加快社会治安视频监控网络建设，加大力度打击各类违法犯罪活动，巩固扩大“长安杯”创建成果。加强食品药品市场监管。加大力度打击制假售假、走私贩私、非法传销。加强安全生产，提高城市消防设施保障水平。强化交通等重点领域安全整治，坚决遏制重特大事故。完善应急事务处置机制，提高应对突发公共事件能力。

十、努力构建为民务实廉洁高效政府

坚持依法行政。深入贯彻落实国务院《依法行政实施纲要》，推动行政决策科学化、法制化、规范化。认真落实市人大及其常委会的决议、决定，及时向人大及其常委会报告工作，自觉接受监督。大力支持人民政协履行政治协商、民主监督、参政议政职能。认真听取民主党派、工商联、无党派人士和人民团体的意见。办好人大代表建议和政协提案。做好行政复议和行政应诉工作。严格执行听证制度和公示制度。圆满完成“五五”普法工作任务。积极开展法治城市、法治县（市、区）和“民主法治示范村（社区）”创建活动。

优化公共服务。在市直机关推行“效能革命”，开展第四轮行政审批项目清理工作，进一步简化审批程序，压减审批时限，提高行政效能。加强市行政服务中心建设，提升“一站式”网上行政审批和电子监察系统服务功能，推进政府部门协同办公和网上联合审批。强化执法监察、廉政监察和效能监察。全面落实政务、厂务和村务公开。

提高执行能力。加强机关作风建设，提高执行能力和抓落实的能力。完善部门年度工作考核目标责任制、行政首长问责制，加强审计工作，加大督查力度，重点问责不作为和乱作为行为。进一步精简会议、文件，严格控制各类评比达标、表彰庆典等活动。

严格廉洁从政。深入实施《建立健全教育、制度、监督并重的惩治和预防腐败体系实施纲要》和《中国共产党党员领导干部廉洁从政若干准则》，加强和完善党风廉政建设责任制，不断完善反腐倡廉制度，大力推进党廉信息公开平台二期建设，坚决查处违法违纪行为，切实纠正损害群众利益的不正之风。各级政府和部门领导要率先垂范，带头廉政勤政，确保安全发展。深入开展市直机关作风评议活动，办好“行风热线”，完善公务员评议考核制度。

建立落实机制。为明确责任，加强监督，量化考核，检查落实，市政府将定期召开专门会议听取专项工作汇报，对重点工作报市委常委会研究，提请人大、政协检查督办，建立责任倒逼机制，确保各项工作的落实。

在全力推进上述各项工作的基础上，今年市政府将集中人力物力财力办好为民惠民十件大事：一是年内完成广佛肇高速公路小湘至德庆至封开段工程可行性研究报告编制工作并尽快报省立项，争取早日让每个山区县都通高速公路；二是年内完成全市25个农贸市场升级改造，为市民提供安全便利的购物场所；三是年内完成牌坊广场改造和星湖栈道一期主体工程，为市民提供更加舒适的休闲环境；四是年内分别完成市第一人民医院新院、市图书馆三分之二主体工程建设，新建300个卫生村和生态文明村，为群众创造良好的文化卫生条件；五是努力缓解端州城区交通拥堵问题，为市民出行提供更加便利的服务；六是年内开工建设市新一中和百花中学，为青少年提供更多的优质学位；七是年内开工建设七星岩东门广场，不断改善景区旅游环境；八是年内全市新建廉租住房278套、经济适用住房346套，继续为城乡困难群众解决住房难问题；九是年内完成景丰联围除险加固达标主体工程，加快城乡安全防洪体系建设；十是年内完成10万农村人口的饮水安全工程建设，切实解决农民饮水难问题。

各位代表，加快推动肇庆实现科学发展新跨越，是时代和人民赋予我们的光荣使命！让我们在中共肇庆市委的正确领导下，深入贯彻落实科学发展观，同心协力，奋力拼搏，真抓实干，开拓创新，努力推动肇庆经济社会又好又快发展，为把我市建设成为未来广东发展的新增长极和能够代表广东科学发展成果的城市而努力奋斗！
